# Nomogram for predicting the overall survival of patients with early‐onset prostate cancer: A population‐based retrospective study

**DOI:** 10.1002/cam4.4694

**Published:** 2022-03-23

**Authors:** Yongtao Hu, Qiao Qi, Yongshun Zheng, Haoran Wang, Jun Zhou, Zongyao Hao, Jialin Meng, Chaozhao Liang

**Affiliations:** ^1^ Department of Urology The First Affiliated Hospital of Anhui Medical University Hefei China; ^2^ Institute of Urology Anhui Medical University Hefei China; ^3^ Anhui Province Key Laboratory of Genitourinary Diseases Anhui Medical University Hefei China; ^4^ Department of General Surgery The First Affiliated Hospital of Anhui Medical University Hefei China

**Keywords:** early‐onset, nomogram, overall survival, prognosis, prostate cancer

## Abstract

**Background:**

The incidence of early‐onset prostate cancer (PCa) has increased significantly over the past few decades. It is necessary to develop a prognostic nomogram for the prediction of overall survival (OS) in early‐onset PCa patients.

**Methods:**

A total of 23,730 early‐onset PCa patients (younger than 55 years old) between 2010 and 2015 in the Surveillance, Epidemiology, and End Results (SEER) database were enrolled for the current study, and randomly separated into the training cohort and the validation cohort. 361 eligible early‐onset PCa patients from The Cancer Genome Atlas‐Prostate Adenocarcinoma (TCGA‐PRAD) cohort were obtained as the external validation cohort. Independent predictors were selected by univariate and multivariate Cox regression analysis, and a prognostic nomogram was constructed for 1‐, 3‐, and 5‐year OS. The accurate and discriminative abilities of the nomogram were evaluated by the concordance index (C‐index), receiver operating characteristic curve (ROC), calibration plot, net reclassification index (NRI), and integrated discrimination improvement (IDI).

**Results:**

Multivariate Cox analysis showed that race, marital status, TNM stage, prostate‐specific antigen, Gleason score, and surgery were significantly associated with poor prognosis of PCa. A nomogram consisting of these variables was established, which had higher C‐indexes than the TNM system (training cohort: 0.831 vs. 0.746, validation cohort: 0.817 vs. 0.752). Better AUCs of the nomogram than the TNM system at 1, 3, and 5 years were found in both the training cohort and the validation cohort. The 3‐year and 5‐year AUCs of the nomogram in the TCGA‐PRAD cohort were 0.723 and 0.679, respectively. The calibration diagram, NRI, and IDI also showed promising prognostic value in OS.

**Conclusions:**

We developed an effective prognostic nomogram for OS prediction in early‐onset PCa patients, which will further assist both the precise clinical treatment and the assessment of long‐term outcomes.

## Graphical abstract

Patients with early‐onset prostate cancer (PCa) have gradually become a distinctive subset under the spotlight. In this study, we developed a prognostic nomogram predicting the overall survival (OS) of early‐onset PCa based on the samples from the Surveillance, Epidemiology and End Results (SEER) database. The nomogram had better predictive accuracy than the traditional TNM staging system, which could guide the clinical treatment and assist the assessment of long‐term outcomes for early‐onset PCa patients.

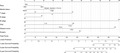

## 1 INTRODUCTION

Prostate cancer (PCa) is the second most common malignant tumor among all malignant tumors in men and ranks first among male genitourinary tumors.^1^ It was estimated that, in 2021, 248,530 new PCa cases were projected to be diagnosed in the United States accounting for 26% of all organically sourced tumors in men.^2^ Since the prostate‐specific antigen (PSA) test started in 1987, the incidence of PCa has been on the rise, especially the early‐onset PCa, which is PCa diagnosed at age ≤55 years old.^3^, ^4^, ^5^ Epidemiological studies showed that the incidence of PCa among young people increased by 5.7‐fold from 1986 to 2008 and the median diagnosis age dropped to 67 years in 2009.^4^ Therefore, patients with early‐onset PCa have become a growing concern in both research and clinical communities.

Young cancer patients are often associated with higher malignancy including advanced cancer stage, poorly differentiated histology, large tumor size, and complex genetic component, which leads to unfavorable prognosis.^6^, ^7^, ^8^ Similar phenomena may also exist in early‐onset PCa. A large population‐based cohort study explored the association between diagnosis age and prognosis in PCa patients and found that young men were at a higher risk of all‐cause death among men with locally advanced PCa than elderly patients.^9^ PCa is a hormone‐dependent disease and different androgen levels are closely related to recurrence and prognosis.^10^ However, there is a significant difference in androgen levels between young and old patients, which could affect the progression of this disease. Meanwhile, compared with old patients, young patients are more likely to receive medical and health care from the outside world.^11^ These findings suggest that there may be different survival outcomes from younger to elderly PCa patients. To optimally evaluate the prognosis and select the appropriate treatment strategy, it is necessary to establish a novel survival assessment model.

The most commonly used decision‐making guidance for clinicians is the TNM staging system.^12^ However, the prognostic value of the TNM staging system is limited due to the lack of accompanying indicators to landscape the overall situation. Nomograms based on the TNM classification system in conjunction with other major clinical parameters have been widely accepted as a visible and accurate model for survival prediction in several tumors.^13^, ^14^, ^15^ Our previous study also applied the nomogram to predict the enlarged prostate volume in benign prostatic hyperplasia and to predict PCa patients' prognosis with clinical features and infiltrated immunocytes.^16^, ^17^ Although some prognostic models for PCa have been published, they are not suitable for assessing the survival of early‐onset PCa patients.^18^, ^19^, ^20^, ^21^ To fill this research gap, we first established a predictive nomogram to predict the OS of early‐onset PCa using data in the SEER database. Additionally, the nomogram was verified and compared with the TNM system to prove its accuracy and reliability. With the help of this nomogram, more optimized clinical decisions will be made, thus helping patients with early‐onset PCa obtain a better prognosis.

## 2 MATERIALS AND METHODS

### 2.1 Data source and patients selection

SEER*Stat software version 8.3.9 was used to extract the latest data from the SEER 18 Registries Research Plus Data (2000–2018) after gaining access to the database (username: 16011‐Nov2020). The clinical features of the TCGA‐PRAD cohort were prior download and used in our team.^22^ In order to obtain complete PSA and Gleason score data and ensure a sufficiently long follow‐up period of enrolled patients, we limited the time of diagnosis between 2010 and 2015. No informed consent or the requirement of the ethics committee prior to the study was required because SEER and TCGA were public databases that did not provide identification information. The inclusion criteria in the current study were as follows: (I) the diagnosis was confirmed by histopathological examination; (II) age of diagnosis ≥18 and ≤55 years; (III) only primary prostate cancer (histology codes: 8410/3) according to the 3rd edition of International Classification of Diseases for Oncology codes; and (IV) complete information on social factors (age, race, marital status), clinicopathological factors (TNM stage, PSA, Gleason score, chemotherapy, radiation, surgery), vital status, and survival time. Nx (unassessable lymph nodes) and Mx (unassessable distant metastasis) cases were excluded from the study. Eligible patients were separated randomly into the training cohort and the validation cohort with an equal number. The detailed search process was shown in Figure S1.

### 2.2 Variable selection

Variables including social factors (age, race, marital status) and clinicopathological factors (TNM stage, PSA, Gleason score, chemotherapy, radiation, surgery) were collected for the current study. All factors were transformed into categorical variables. Age was separated as <50 years and ≥50 years. Race was divided into Black, White, and other. Marital status was categorized into four classes: married, separated or divorced, single, and widowed. The definition of clinical stage was based on the 8th edition of American Joint Committee on Cancer (AJCC) TNM staging system. According to the risk classification of PCa, PSA was divided into four levels: <4 ng/ml (normal), 4–10 ng/ml (low risk), 10–20 ng/ml (medium risk), and >20 ng/ml (high risk).^23^ Gleason score was also classified as ≤6, 7, and ≥8, of which Gleason score 7 was further divided into Gleason 3 + 4 and Gleason 4 + 3. Chemotherapy, radiation, and surgery were categorized according to whether the patient received the treatment.

### 2.3 Statistical analysis

Statistical analyses were completed using R software version 4.1.1. All categorical variables were presented as frequencies and proportions. The assumption of proportional hazards was evaluated by the Schoenfeld residual test.^24^ Univariate Cox regression analysis and subsequent multivariate Cox regression analysis were performed to reveal the OS‐related independent prognostic factors.^25^, ^26^, ^27^ A prognostic nomogram for predicting OS probabilities was developed according to the final included factors. Kaplan–Meier (KM) plots and log‐rank tests were conducted to generate survival curves and identify significant differences in survival rates in the different subgroups. The C‐index and area under the ROC curve (AUC) were calculated to compare the discriminative ability between our nomogram and conventional TNM staging system.^28^ The C‐index and AUC >0.7 were considered to be sufficiently discriminative. The accuracy of the nomogram was tested by calibration plots with 1000 bootstraps resamples.^29^ In addition, NRI and IDI were calculated to compare the clinical usefulness between the nomogram and TNM staging system.^30^ A two‐sided *P* value <0.05 was regarded as statistically significant.

## 3 RESULTS

### 3.1 Patient characteristics

We identified 256,475 PCa patients from the SEER database during 2010–2015. According to the aforementioned inclusion criteria, a full analysis cohort of 23,730 eligible patients with early‐onset PCa was eventually enrolled in this study. Among all patients, 11,866 patients were assigned to the training cohort and the rest to the validation cohort. A total of 361 eligible early‐onset PCa patients were collected from the TCGA‐PRAD cohort. Counts and proportions on the characteristics of eligible early‐onset PCa patients obtained from the SEER database were demonstrated in Table 1. The median age in the training cohort and validation cohort was 52 (range, 34–55) years old and 52 (range, 29–55) years old, respectively. In the full analysis cohort, White patients accounted for 71.2% of the cases, much larger than Black patients and other races. The majority of the patients (70.7%) were married. With regard to the TNM stage, most of the patients had T2 stage (57.2%), N0 stage (96.3%), and M0 stage (97.4%). More than 50% of the patients were diagnosed with a PSA level of 4–10 ng/ml, and a similar phenomenon was observed in Gleason score ≤6. Concerning treatment, radiation was received by 5214 patients (22.0%), and only 199 patients (0.8%) were treated with chemotherapy. We also noticed that approximately 64.9% of patients had surgery, which was considered an important factor in prognosis.

**TABLE 1:** Counts and proportions on the characteristics of eligible patients with early‐onset prostate cancer

Variables	Level	All patients (*n* = 23,730)		Training cohort (*n* = 11,866)		Validation cohort (*n* = 11,864)	
		*N*	%	*N*	%	*N*	%
Age	<50	5371	22.6	2685	22.6	2686	22.6
	50–55	18,359	77.4	9181	77.4	9178	77.4
Race	Black	5967	25.1	2937	24.8	3030	25.5
	White	16,891	71.2	8494	71.6	8397	70.8
	Other	872	3.7	435	3.7	437	3.7
Marital status	Married	16,786	70.7	8439	71.1	8347	70.4
	Separated or Divorced	2245	9.5	1094	9.2	1151	9.7
	Single	4517	19.0	2258	19.0	2259	19.0
	Widowed	182	0.8	75	0.6	107	0.9
Year of diagnosis	2010	4722	19.9	2342	19.7	2380	20.1
	2011	4593	19.4	2366	19.9	2227	18.8
	2012	3963	16.7	1923	16.2	2040	17.2
	2013	3607	15.2	1753	14.8	1854	15.6
	2014	3422	14.4	1689	14.2	1733	14.6
	2015	3423	14.4	1793	15.1	1630	13.7
T stage	T1	6589	27.8	3280	27.6	3309	27.9
	T2	13,569	57.2	6780	57.1	6789	57.2
	T3	3374	14.2	1719	14.5	1655	13.9
	T4	198	0.8	87	0.7	111	0.9
N stage	N0	22,847	96.3	11,416	96.2	11,431	96.4
	N1	883	3.7	450	3.8	433	3.6
M stage	M0	23,116	97.4	11,567	97.5	11,549	97.3
	M1	614	2.6	299	2.5	315	2.7
PSA	<4	4368	18.4	2217	18.7	2151	18.1
	4–10	14,772	62.3	7358	62.0	7414	62.5
	10–20	2611	11.0	1309	11.0	1302	11.0
	>20	1979	8.3	982	8.3	997	8.4
Gleason score	<=6	12,140	51.2	6083	51.3	6057	51.1
	7 (3 + 4)	6727	28.3	3323	28.0	3404	28.7
	7 (4 + 3)	2310	9.7	1162	9.8	1148	9.7
	>=8	2553	10.8	1298	10.9	1255	10.6
Chemotherapy	Yes	199	0.8	95	0.8	104	0.9
	No/Unknown	23,531	99.2	11,771	99.2	11,760	99.1
Radiation	Yes	5214	22.0	2618	22.1	2596	21.9
	No/Unknown	18,516	78.0	9248	77.9	9268	78.1
Surgery	Yes	15,404	64.9	7746	65.3	7658	64.5
	No	8326	35.1	4120	34.7	4206	35.5
Vital status	Alive	22,479	94.7	11,250	94.8	11,229	94.6
	Dead	1251	5.3	616	5.2	635	5.4

Abbreviation: PSA, prostate‐specific antigen.

### 3.2 Selection of prognostic factors

**FIGURE 1:**
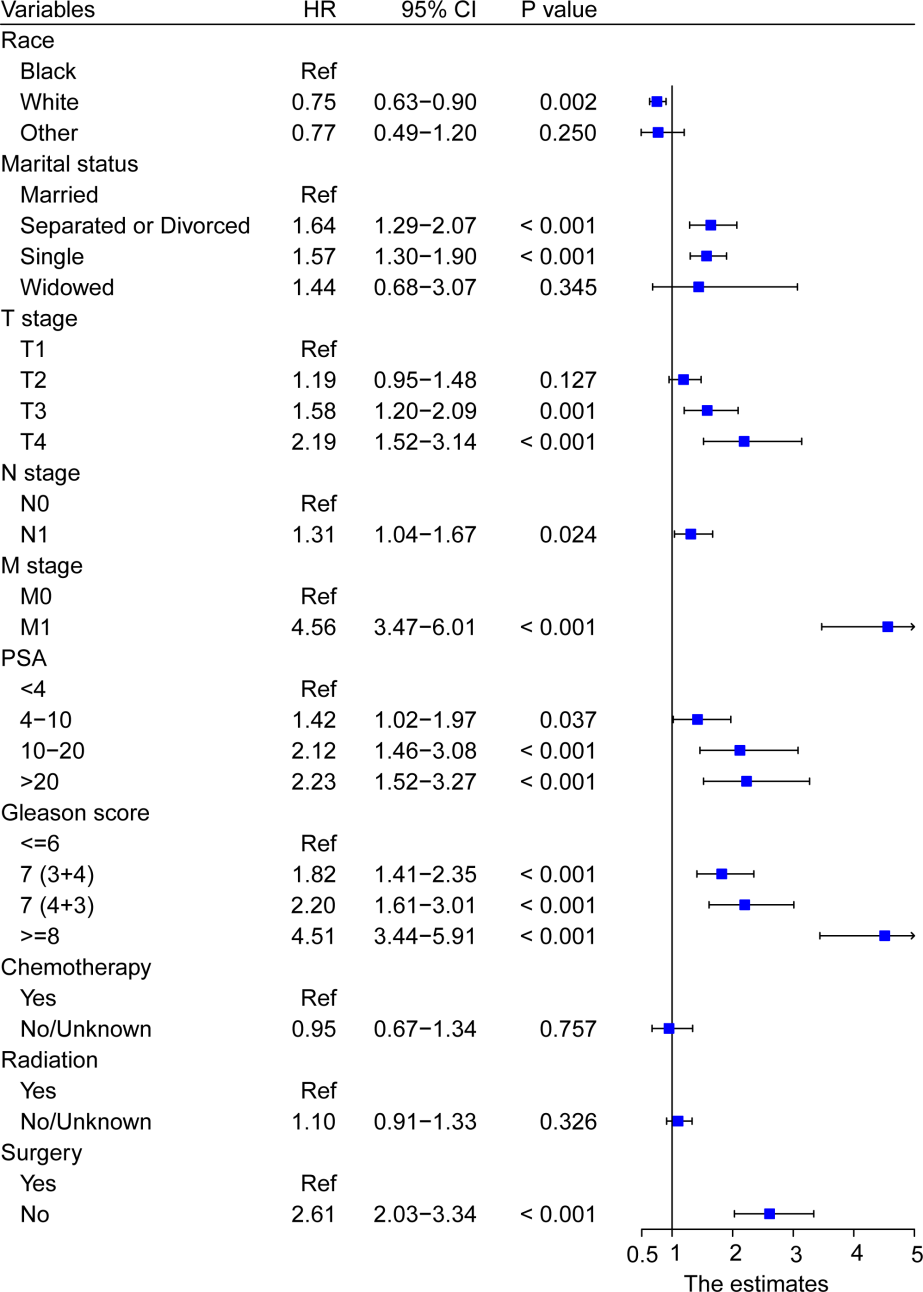
Forest plot showing the results of multivariate Cox regression analysis in clinical parameter subgroups

Apart from diagnosis year being unsuitable for inclusion in the predictive analysis, all variables in the training cohort were entered into the univariate analyses, and the results revealed that race, marital status, TNM stage, PSA, Gleason score, chemotherapy, radiation, and surgery had a significant association with OS (Table 2). Multivariate analysis included these positive variables and finally showed that race, marital status, T stage, N stage, M stage, PSA, Gleason score, and surgery were statistically significant factors for OS (all *p* < 0.05), while radiation and chemotherapy showed no prognostic efficacy (Figure 1). No major violations of the proportional hazards assumption were detected.

**TABLE 2:** Univariate and multivariate Cox analysis of the training cohort on overall survival

Variables	Level	Univariate analysis			Multivariate analysis		
		HR	95% CI	*p* value	HR	95% CI	*p* value
Age	<50	Reference			‐	‐	‐
	50–55	1.21	0.99–1.48	0.058	‐	‐	‐
Race	Black	Reference			Reference		
	White	0.58	0.49–0.68	<0.001^*^	0.75	0.63–0.90	0.002^*^
	Other	0.67	0.43–1.04	0.074	0.77	0.49–1.20	0.250
Marital status	Married	Reference			Reference		
	Separated or Divorced	2.56	2.03–3.22	<0.001^*^	1.64	1.29–2.07	<0.001^*^
	Single	2.87	2.41–3.42	<0.001^*^	1.57	1.30–1.90	<0.001^*^
	Widowed	2.76	1.31–5.85	0.008^*^	1.44	0.68–3.07	0.345
T stage	T1	Reference			Reference		
	T2	0.52	0.44–0.63	<0.001^*^	1.19	0.95–1.48	0.127
	T3	1.20	0.96–1.49	0.112	1.58	1.20–2.09	0.001^*^
	T4	11.08	7.97–15.40	<0.001^*^	2.19	1.52–3.14	<0.001^*^
N stage	N0	Reference			Reference		
	N1	9.25	7.63–11.21	<0.001^*^	1.31	1.04–1.67	0.024^*^
M stage	M0	Reference			Reference		
	M1	31.04	25.99–37.08	<0.001^*^	4.56	3.47–6.01	<0.001^*^
PSA	<4	Reference			Reference		
	4–10	1.69	1.22–2.34	0.002^*^	1.42	1.02–1.97	0.037^*^
	10–20	4.13	2.88–5.92	<0.001^*^	2.12	1.46–3.08	<0.001^*^
	>20	17.18	12.42–23.77	<0.001^*^	2.23	1.52–3.27	<0.001^*^
Gleason score	<=6	Reference			Reference		
	7 (3 + 4)	1.98	1.54–2.53	<0.001^*^	1.82	1.41–2.35	<0.001^*^
	7 (4 + 3)	3.12	2.32–4.19	<0.001^*^	2.20	1.61–3.01	<0.001^*^
	> = 8	13.97	11.33–17.23	<0.001^*^	4.51	3.44–5.91	<0.001^*^
Chemotherapy	Yes	Reference			Reference		
	No/Unknown	0.07	0.05–0.10	<0.001^*^	0.95	0.67–1.34	0.757
Radiation	Yes	Reference			Reference		
	No/Unknown	0.50	0.43–0.59	<0.001^*^	1.10	0.91–1.33	0.326
Surgery	Yes	Reference			Reference		
	No	3.72	3.15–4.39	<0.001^*^	2.61	2.03–3.34	<0.001^*^

Abbreviations: CI, confidence interval; HR, hazard ratio; PSA, prostate‐specific antigen.

^*^ *p* < 0.05.

**FIGURE 2:**
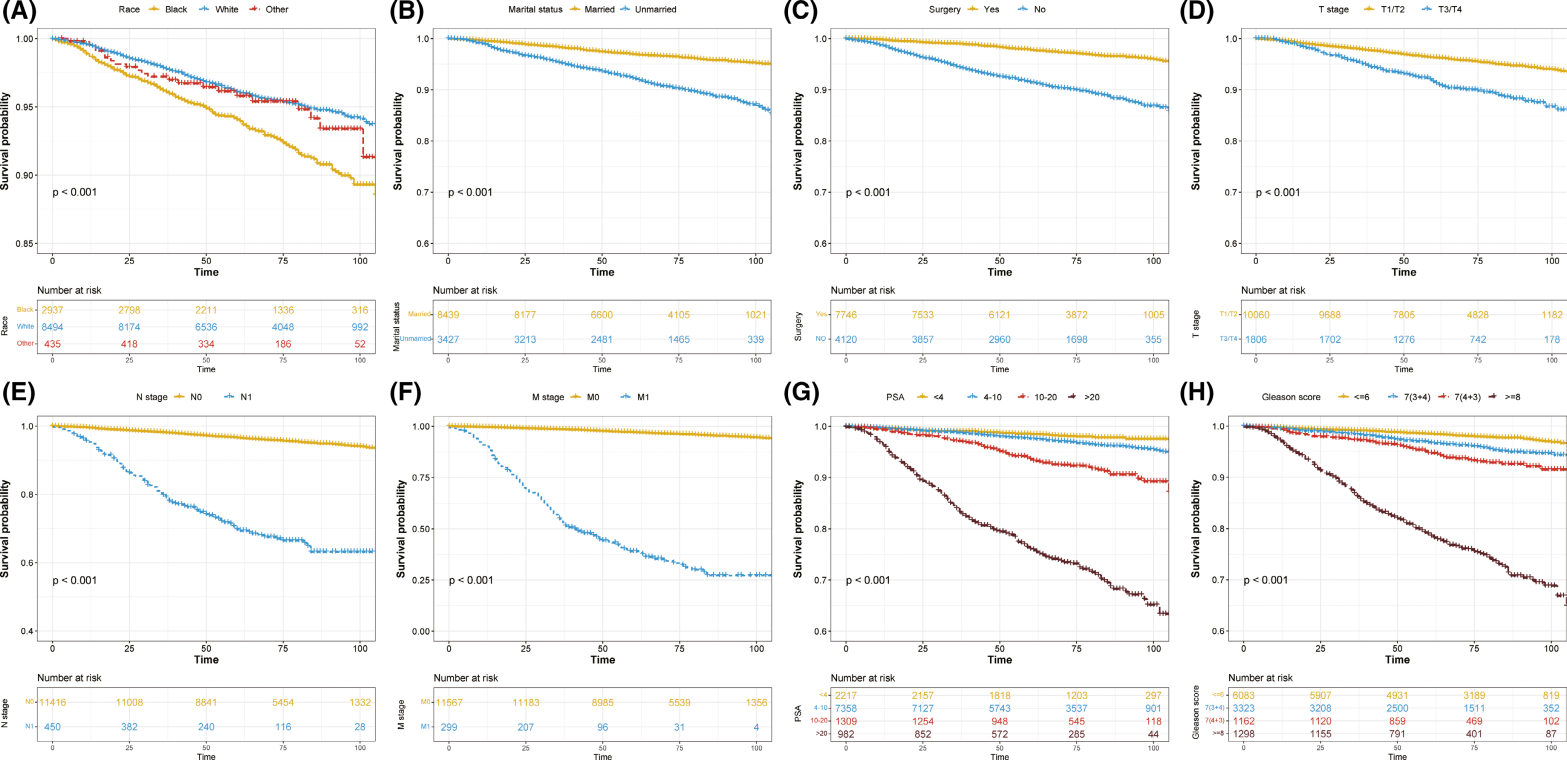
Kaplan–Meier curves present the diverse overall survival in early‐onset prostate cancer patients stratified by different clinical parameters. (A) race; (B) marital status; (C) surgery; (D) T stage; (E) N stage; (F) M stage; (G) PSA; (H) Gleason score. PSA, prostate‐specific antigen

The follow‐up time of early‐onset PCa patients in the current study ranged from 0 to 107 months, and the median value was 72 months. To further assess the survival outcome of different subgroups, we performed KM survival analysis in the training cohort. OS of patients who were Whites was better than that of Blacks (*p* < 0.001; Figure 2A, Figure S2A) while no significant survival difference was observed either between Blacks and other races (*p* = 0.074, Figure S2B) or between Whites and other races (*p* = 0.49, Figure S2C). Compared with unmarried patients, the OS of married patients was significantly increased (*p* < 0.001; Figure 2B). With regard to surgery, patients who received surgery tended to have significantly increased OS (*p* < 0.001; Figure 2C). As shown in Figure 2D‐F, higher TNM stage was related to worse OS (*p* < 0.001). A similar tendency was also found in Figure 2G‐H, which indicated that higher PSA and Gleason score could worsen the prognosis of patients (*p* < 0.001).

### 3.3 Construction and validation of nomogram

**FIGURE 3:**
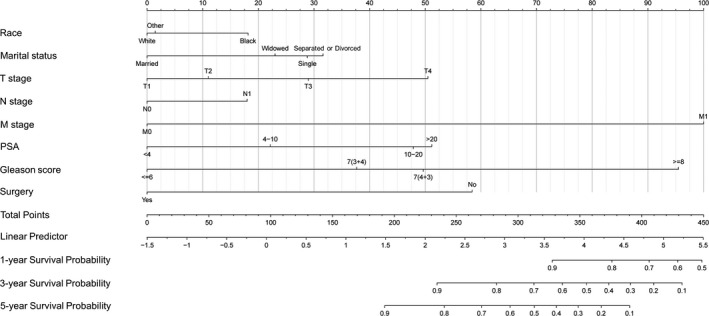
Newly defined nomogram for the overall survival prediction of early‐onset prostate cancer patients

A nomogram using the risk factors obtained from multivariate Cox regression analysis was constructed to predict the 1‐, 3‐, and 5‐year OS of patients with early‐onset PCa (Figure 3). Each factor has its corresponding score value on the points scale and the maximum score is 100 points. A total score could be obtained by adding the scores of all selected factors, and then the corresponding 1‐, 3‐, and 5‐year OS could be estimated by the nomogram scoring system (Table 3). The nomogram revealed that M stage was the most influential prognostic factor, closely followed by Gleason score. In addition, surgery, PSA, T stage, marital status, and race made a moderate contribution to the survival outcome, while N stage played minor roles.

**TABLE 3:** Nomogram scoring system

Variables	Points	Variables	Points	Variables	Points
Race		T stage		PSA	
Black	18	T1	0	<4	0
White	0	T2	11	4–10	22
Other	1	T3	29	10–20	48
Marital status		T4	50	>20	51
Married	0	N stage		Gleason score	
Separated or Divorced	32	N0	0	<=6	0
Single	29	N1	18	7 (3 + 4)	38
Widowed	23	M stage		7 (4 + 3)	50
Surgery		M0	0	> = 8	96
Yes	0	M1	100		
No	58				

1‐Year OS probability	Points	3‐Year OS probability	Points	5‐Year OS probability	Points
0.9	328	0.9	235	0.9	192
0.8	376	0.8	283	0.8	240
0.7	406	0.7	313	0.7	270
0.6	429	0.6	336	0.6	294
0.5	449	0.5	356	0.5	313
		0.4	373	0.4	331
		0.3	391	0.3	349
		0.2	410	0.2	367
		0.1	433	0.1	390

Abbreviations: OS, overall survival; PSA, prostate‐specific antigen.

**FIGURE 4:**
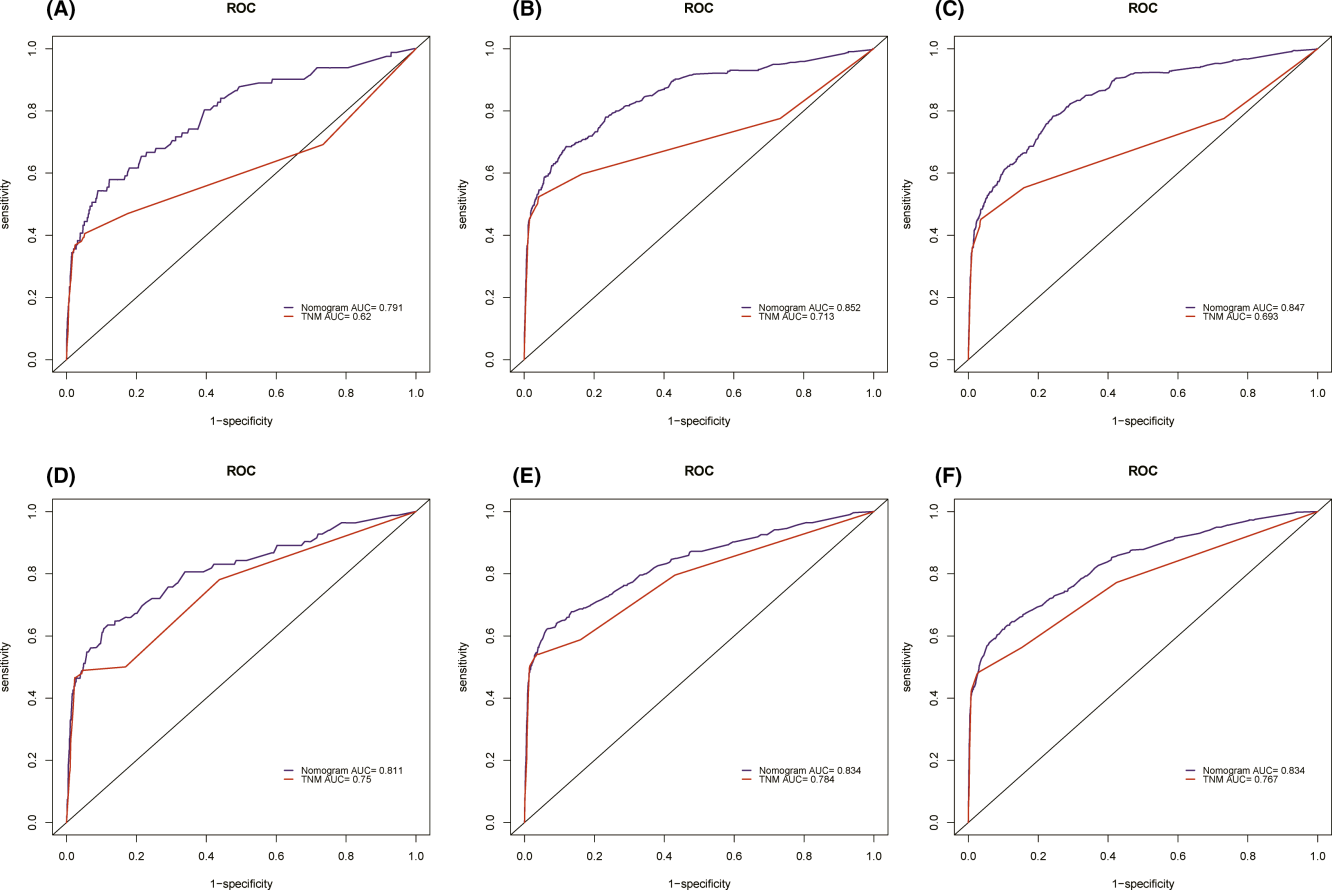
Comparison of the prognostic value of the TNM system and newly constructed nomogram by 1‐, 3‐, and 5‐year ROC curves in the training cohort (A–C) and the validation cohort (D–F)

**FIGURE 5:**
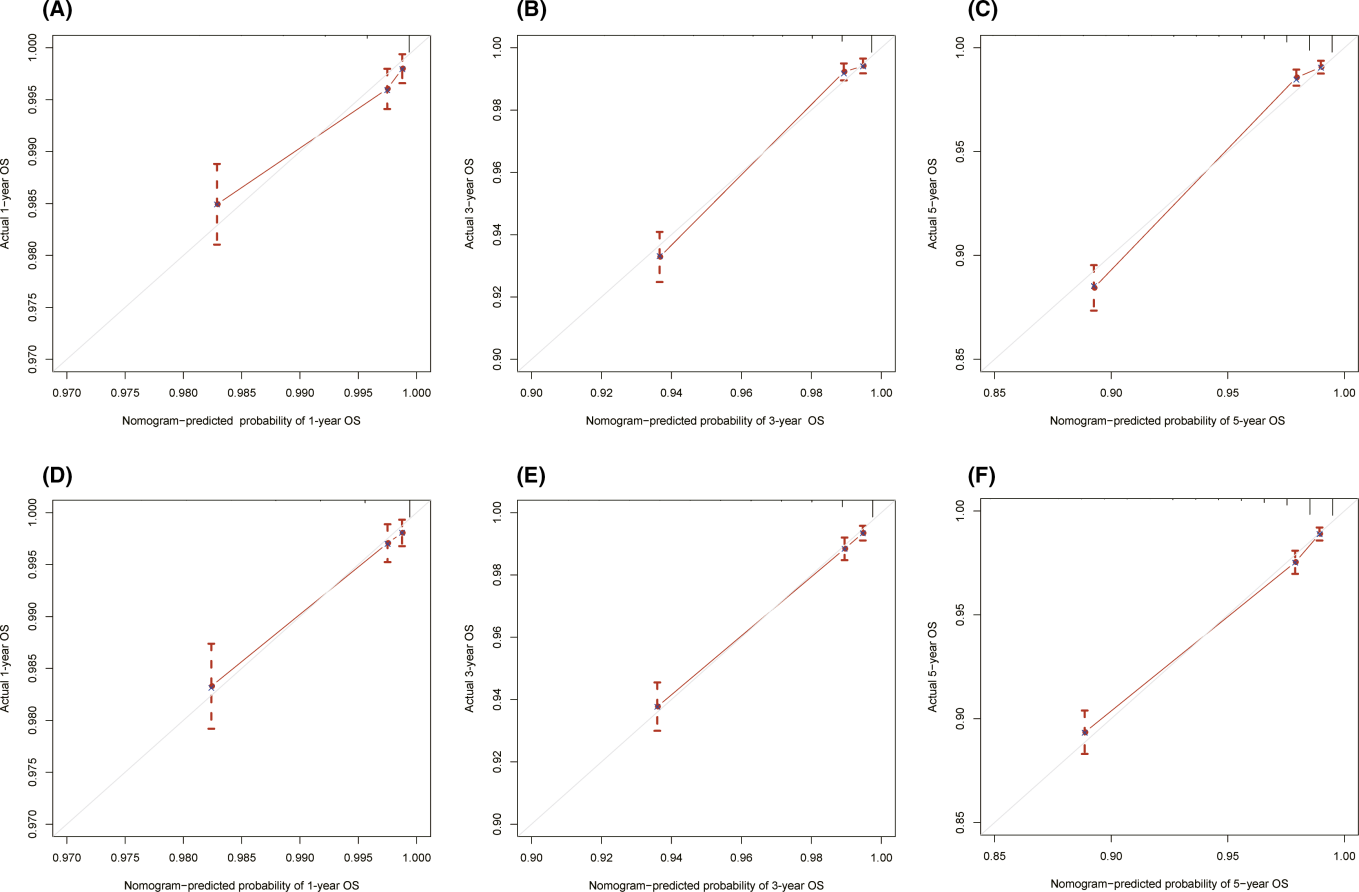
Calibration curves of the nomogram for 1‐, 3‐, and 5‐year overall survival prediction in the training cohort (A–C) and the validation cohort (D–F). The x‐axis represents the nomogram‐predicted probability of overall survival, and the y‐axis represents the actual probability of overall survival. The diagonal 45‐degree line in the calibration plot indicates higher prediction accuracy

The C‐index of the newly constructed nomogram was higher than that of the TNM system (training cohort: 0.831 vs. 0.746; validation cohort: 0.817 vs. 0.752), which indicated that the model had great discriminative ability in predicting the prognosis of early‐onset PCa. As shown in Figure 4A‐F, the nomogram had significantly higher AUC values than TNM system in the training cohort (1‐year: 0.791 vs. 0.620, 3‐year: 0.852 vs. 0.713, 5‐year: 0.847 vs. 0.693) while better AUC values were also found in the validation cohort (1‐year: 0.811 vs. 0.750, 3‐year: 0.834 vs. 0.784, 5‐year: 0.834 vs. 0.767). Based on the total points of Race, TNM stage, PSA, Gleason score, and surgery, the AUC values of the nomogram for 3‐year and 5‐year in the TCGA‐PRAD cohort were 0.723 and 0.679, respectively (Figure S3). The excellent accuracy of the prediction value of the nomogram was also assessed by the calibration curves and a preferable consistency between the nomogram predicted and actual observed values was presented in both cohorts (Figure 5A‐F). The NRI and IND values further indicated that the nomogram had higher predictive power for OS than the TNM system (Table S1).

## 4 DISCUSSION

Patients with early‐onset PCa have gradually become a distinctive subset among PCa patients due to the significantly increased incidence.^31^ Accurately and effectively predicting the prognosis of cancer patients is of great significance for clinical treatment and guideline formulation.^32^, ^33^ Patient prognosis is reflected not only by several pathological indicators included in the TNM classification system but also by social and other clinicopathological factors. Therefore, with the concept of precision medicine proposed, the TNM classification system was unable to meet the needs of clinicians.^34^ Here, we first constructed an accurate nomogram based on a large retrospective case series to predict the OS of early‐onset PCa patients. Quantitative indicators provided by the nomogram could assist clinicians in improving the prognosis of patients.

Two social factors (race, marital status) and six clinicopathological factors(TNM stage, PSA, Gleason score, surgery) were identified as independent prognostic factors for OS in patients with early‐onset PCa. We then integrated the eight factors into a nomogram in the training cohort to predict 1‐, 3‐, and 5‐year OS. The C‐indexes and AUCs of the nomogram in both two cohorts were significantly higher than those of the TNM system, and the calibration curves were closely matched to the ideal standard line. Furthermore, NRI and IDI analyses also indicated that the nomogram had higher predictive power than the conventional TNM system. There are several reasons to explain the superior results of this study. First, our nomogram incorporated social and more clinicopathological factors than the traditional TNM system in assessing patient prognosis. The TNM staging system only took clinical TNM stage into account but ignored the significant impact of PSA, Gleason score, and surgery on survival outcomes. As shown in the nomogram, Gleason score, surgery, and PSA were the second, third, and fourth largest contributors to prognosis after M stage. Second, the TNM staging system was not quantitative enough, and it did not reflect the different effects of TNM stage on prognosis. Instead, in this study, we used risk scores to reflect the weight of different factors on the outcome and presented them in a quantitative and graphical tool, which facilitated the clinician's work. Third, the patients in the study were more representative because they were screened based on strict criteria, therefore our nomogram had more clinical practicability for patients with early‐onset PCa.

Age has been generally recognized as a crucial prognostic factor for patient survival.^35^, ^36^, ^37^ Elderly patients are often associated with age‐related comorbidities including cardiovascular diseases, neurological disorders, chronic pulmonary diseases, and metabolic dysfunction, all of which could worsen the prognosis and contribute to mortality. However, different from other studies, our study failed to identify age as a prognostic factor for OS. Consistent with our findings, this phenomenon was also observed in early‐onset gastric cancer.^38^ The mechanism for the observed difference was that elderly patients had a significantly higher proportion in these cohorts than in ours. Patients included in this study all had early‐onset PCa, which was defined as age ≤55 years old. People at this age were at their high levels of physical condition and social support, and the physical and social differences between <50 years and ≥50 years were not obvious. With the development of surgery and other adjunctive treatments, the OS rate for PCa patients has improved obviously, but it is not synchronized among different races with greater mortality in African Americans.^39^ This was consistent with our research results. In KM survival analysis, we analyzed the survival of different races and eventually found that Blacks had a lower OS rate than Whites. Genetic susceptibility and environmental factors were tightly linked to the occurrence and progression of PCa. In American society, African Americans experience a higher PCa rate, obtain reduced financial resources, have lower social support, and receive more unequal health care when compared with other races.^40^ Therefore, the government and citizens should take necessary measures to improve the OS of African Americans. In addition to the abovementioned social factors, marital status was also a predictor of survival in patients with early‐onset PCa. Similar to many previous studies,^41^, ^42^, ^43^ married PCa patients presented with more satisfying survival than unmarried patients. The proposed reason for explaining this phenomenon was that married patients were more likely to obtain financial support and emotional pillars from spouses. The heavy financial burden and the accompanying emotional disorders could lead to poor outcomes in separated, divorced, single, and widowed patients.

PSA, a protease consisting of 237 amino acids, has become an important tumor marker for the diagnosis, staging, therapeutic effect monitoring, and prognosis of PCa. High levels of PSA in PCa patients often indicate tumor recurrence or metastasis,^44^, ^45^ which could explain why patients with high PSA, especially those in the high‐risk subgroup, had unfavorable prognoses in our study. Unlike PSA, Gleason score is a tool to evaluate the malignancy of PCa according to the degree of gland differentiation and tumor growth pattern. In the Gleason grading system, tumors can be divided into primary and secondary grading areas according to the proportion of tumors with different morphological structures. We divided Gleason score into three risk levels, and Gleason score 7 was further divided into Gleason 3 + 4 and Gleason 4 + 3 due to the various prognoses between the two subgroups. Previous studies reported that Gleason 4 + 3 had a higher rate of biochemical recurrence and distant metastasis than Gleason 3 + 4.^46^, ^47^ Consistent with these reports, our study confirmed that Gleason 4 + 3 contributed more to poor prognosis and had a lower OS rate than Gleason 3 + 4. Large‐scale statistical research performed by Zhou et al. studied the different prognoses of each Gleason subgroup detailedly and created a new Gleason survival grading system.^48^ In their survival grading system, grade 3 with Gleason 4 + 3 did have higher overall and cancer‐specific death than grade 2 with Gleason 3 + 4. Moreover, grade 4 with Gleason 4 + 4/3 + 5, grade 5 with Gleason 5 + 3/4 + 5, and grade 6 with Gleason 5 + 4/5 + 5 had significantly worse OS and cancer‐specific survival than grade 3 with Gleason 4 + 3, which was in line with our research findings.

It is worth noting that radiation and chemotherapy were not identified as independent predictors in multivariate analysis. Patients who receive radiation or chemotherapy often have large tumors or distant metastases.^49^, ^50^, ^51^, ^52^ The effect of radiotherapy and chemotherapy on survival in these PCa patients was not statistically significant compared with that in relatively early‐stage PCa patients who did not receive radiotherapy and chemotherapy. Noteworthy, M stage and Gleason score contributed more to poor prognosis than surgery. This is because the higher the Gleason score is, the higher the malignancy of the tumor, and the lower the possibility of survival.^48^, ^53^ Kweldam et al. found that Gleason score ≥7 was associated with a high rate of lymph node and distant metastasis.^54^ Patients with distant metastases lose the chance of radical surgery and often have a poor prognosis, which suggests that early diagnosis and treatment have important significance for the survival of PCa patients.

Some prognostic models for the survival of PCa patients have been reported but almost all of them were nomograms of patients with castration‐resistant PCa.^55^, ^56^, ^57^, ^58^ It is worth mentioning that the current study provided the first nomogram to predict the clinical outcome of early‐onset PCa patients. Of course, the limitations should also be considered. Primarily, the unknown data were deleted, which might lead to selection bias and affect the accuracy and clinical practicability of our nomogram; therefore, validation based on other cohorts is necessary to confirm our conclusions. In addition, the detailed radiation and chemotherapy data and the reasons why a proportion of people did not undergo surgery were unclear, all of which require further exploration in future studies. Moreover, smoking, comorbidities, surgical margin status, endocrine therapy, and other potential prognostic factors were not mentioned in the SEER database and were not included in the nomogram, which might impact the accuracy of the nomogram. Finally, the included patients were all registered in the U.S.A; therefore, the conclusions should be interpreted with caution when applying the nomogram in other countries.

## 5 CONCLUSION

Race, marital status, TNM stage, PSA, Gleason score, and surgery were independent prognostic factors for the OS of early‐onset PCa patients. Although some limitations existed in the study, the nomogram based on these factors presented superior accuracy and applicability to predict the clinical outcome of early‐onset PCa patients, which could help the optimization of clinical decision‐making.

## CONFLICT OF INTEREST

None declared.

## AUTHOR CONTRIBUTIONS

Yongtao Hu and Qiao Qi designed this study. Qiao Qi, Yongshun Zheng, and Haoran Wang collected the data. Yongtao Hu, Jun Zhou, and Zongyao Hao integrated and analyzed the data. Yongtao Hu and Qiao Qi wrote this manuscript. Jialin Meng and Chaozhao Liang edited and revised the manuscript. All authors approved the final manuscript.

## ETHICS APPROVAL

The data of this research were obtained from the public databases and no ethical approval was required.

## Supporting information


Figure S1

Figure S2

Figure S3

Table S1

## Data Availability

All data used in this study can be acquired from the SEER database (https://seer.cancer.gov/) and the TCGA database (https://tcga‐data.nci.nih.gov/tcga).
