# Supplementary figures and images for: Nomogram for predicting the overall survival of patients with early‐onset prostate cancer: A population‐based retrospective study
Source: Cancer Med. 2022 Mar 23;11(17):3260–71. doi: 10.1002/cam4.4694 (PMC9468440; doi:10.1002/cam4.4694)

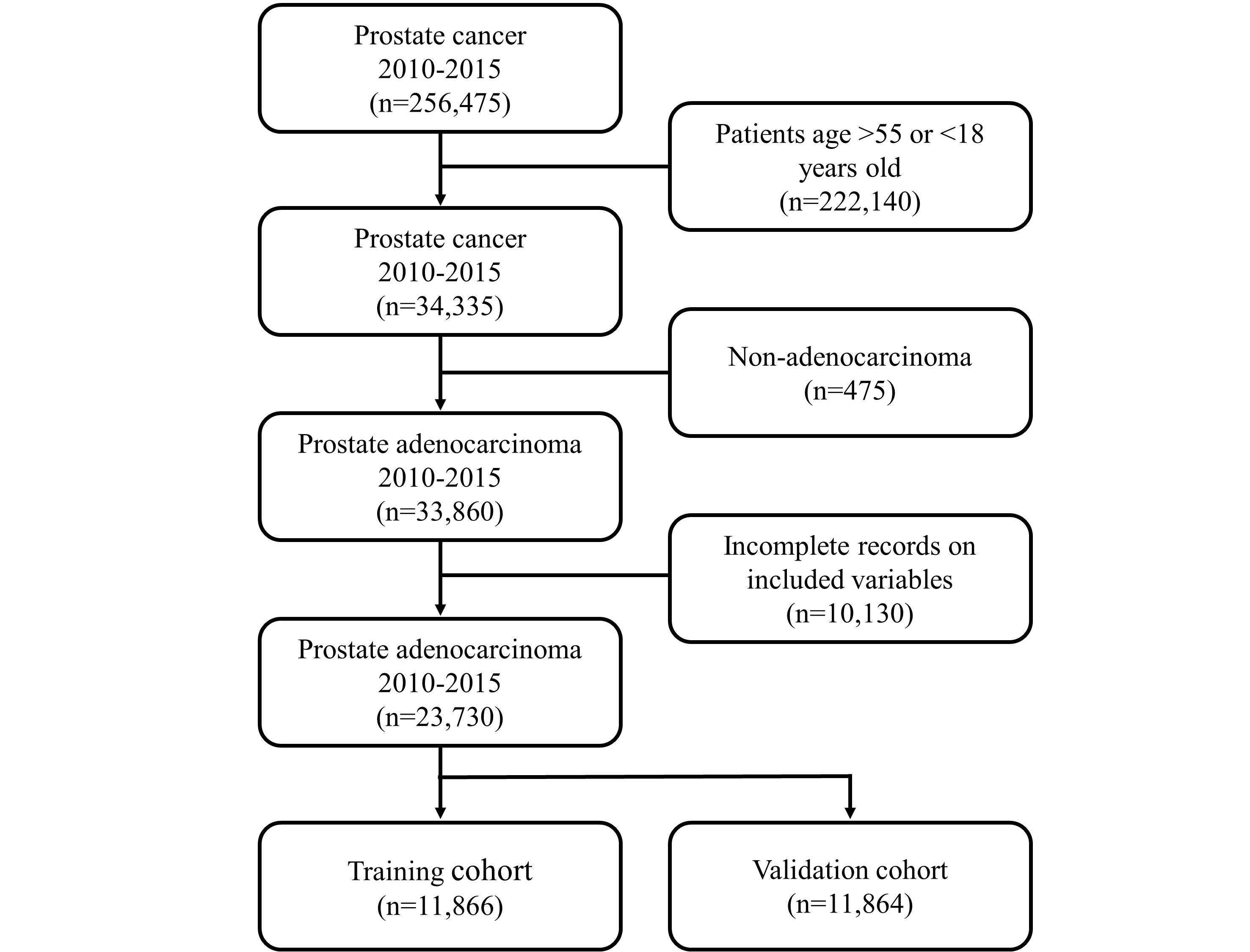

Supplement: Supplementary file 1 — Figure S1 [file CAM4-11-3260-s002.tif]

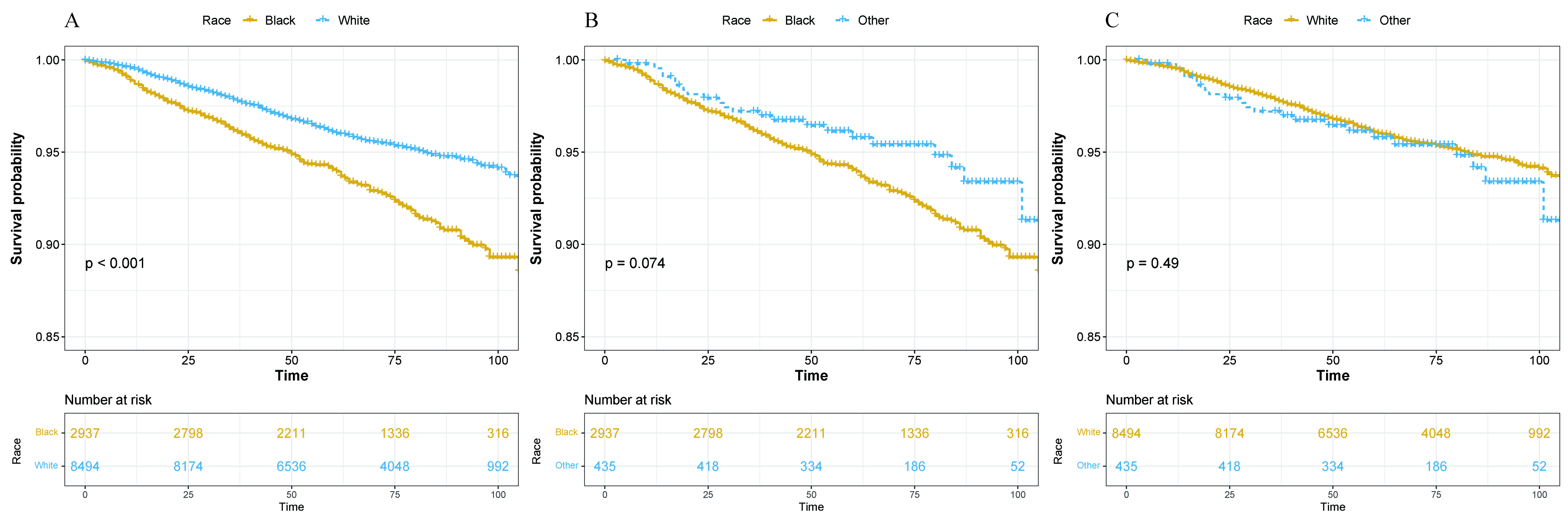

Supplement: Supplementary file 2 — Figure S2 [file CAM4-11-3260-s003.tif]

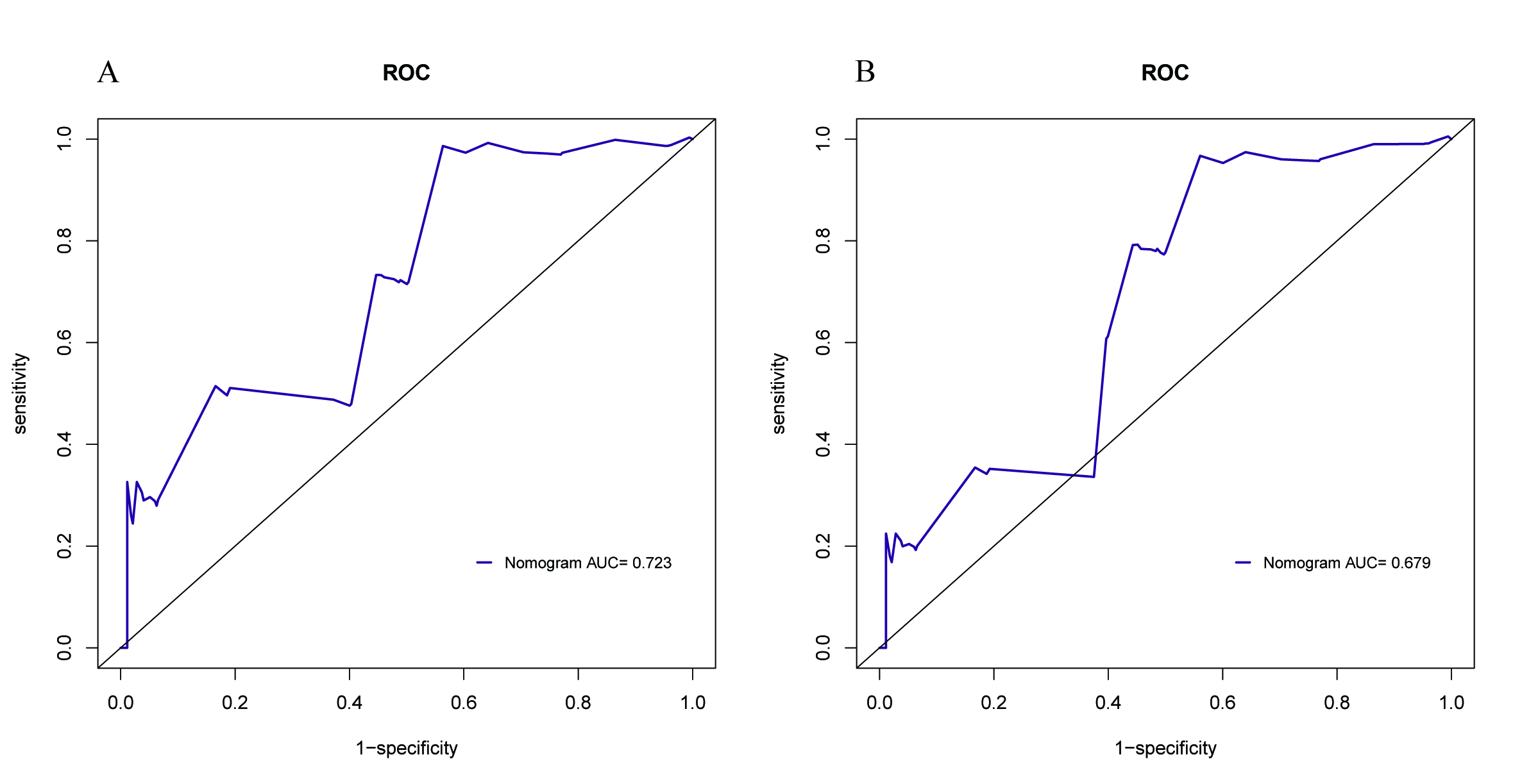

Supplement: Supplementary file 3 — Figure S3 [file CAM4-11-3260-s004.tif]
