# Supplementary material for: Nomogram for predicting the overall survival of patients with early‐onset prostate cancer: A population‐based retrospective study
Source: Cancer Med. 2022 Mar 23;11(17):3260–71. doi: 10.1002/cam4.4694 (PMC9468440; doi:10.1002/cam4.4694)
Supplement: Supplementary file 4 — Table S1 [file CAM4-11-3260-s001.docx]

Table S1. NRI and IDI of the nomogram and TNM staging system in the training cohort.

| Index | Estimate | 95% CI | *P* value |
| --- | --- | --- | --- |
| NRI (vs. the TNM staging system) |  |  |  |
| For 1-year OS | 0.427 | 0.294-0.546 | < 0.01^*^ |
| For 3-year OS | 0.497 | 0.434-0.545 | < 0.01^*^ |
| For 5-year OS | 0.497 | 0.406-0.543 | < 0.01^*^ |
| IDI (vs. the TNM staging system) |  |  |  |
| For 1-year OS | 0.056 | 0.035-0.085 | < 0.01^*^ |
| For 3-year OS | 0.190 | 0.153-0.226 | < 0.01^*^ |
| For 5-year OS | 0.222 | 0.189-0.253 | < 0.01^*^ |

CI, confidence interval; NRI, net reclassification index; IDI, integrated discrimination improvement; OS, overall survival; ^*^, *P* < 0.05.
